# Tissue-specific alteration of gene expression and function by RU486 and the GeneSwitch system

**DOI:** 10.1038/s41514-019-0036-8

**Published:** 2019-05-21

**Authors:** Maricela Robles-Murguia, Liam C. Hunt, David Finkelstein, Yiping Fan, Fabio Demontis

**Affiliations:** 1 Division of Developmental Biology, Department of Developmental Neurobiology, St. Jude Children’s Research Hospital, Memphis, TN 38105 USA; 2 Department of Computational Biology, St. Jude Children’s Research Hospital, Memphis, TN 38105 USA

**Keywords:** Genome, Medical research

## Abstract

The GeneSwitch (GS) is a modified Gal4/UAS system, whereby transgene expression is induced in *Drosophila* by adding the drug RU486 to food. The GS system is routinely used in *Drosophila* aging and behavioral studies to avoid confounding effects related to genetic background mutations. Here, we report transcriptional and functional defects that are induced by RU486 in a stock- and tissue-dependent manner, such as defects in flight and mitochondrial gene expression. In addition to including proper controls, our findings suggest that context-specific side effects induced by RU486 should be considered in the experimental design and when interpreting the observed phenotypes.

## Introduction

GeneSwitch (GS) is a modified Gal4/UAS system, whereby transgene expression is induced in *Drosophila* by adding the drug RU486 (mifepristone) to food. This drug-inducible system is most widely used in *Drosophila* behavioral, metabolic, and aging studies to exclude the contribution of cytoplasmic and genetic background mutations to observed phenotypes.^1,2^ Its widespread use has been sustained by the apparent lack of major caveats and the extensive knowledge that has been gained about this system since its introduction in 2001.^1,2^ However, a few concerns have been raised about the GS system, including substantial leakiness of some GS-Gal4 drivers and effects of RU486 under specific dietary conditions.^3–6^ Altogether, the knowledge gained from these studies has led to improved experimental designs and avoidance of confounding effects derived from the use of RU486 under specific circumstances.^3–6^ On this basis, to improve the usage of the GS system in skeletal muscle, we have generated a new muscle-specific GS-Gal4 driver and examined its efficacy in this tissue.

## Results

We have generated a new GeneSwitch Gal4 driver for drug-induced transgene expression in flight muscles, *Act88F-GS* (see the “Methods” section). Compared with Mhc-GS-Gal4, which is leaky,^3^ we find that *Act88F-GS* does not drive any meaningful *foxo* and *mCherry* transgenic expression in the absence of RU486 (Supplementary Figure 1). Moreover, it drives expression specifically in thoraces, as expected based on the flight muscle-restricted expression of *Act88F*. Consistent with previous studies with skeletal muscle-specific constitutive Gal4 drivers,^7^ we find that also RU486-induced muscle-specific overexpression of *foxo* with *Act88F-GS* extends the life span (Supplementary Figure 1).

**Fig. 1:**
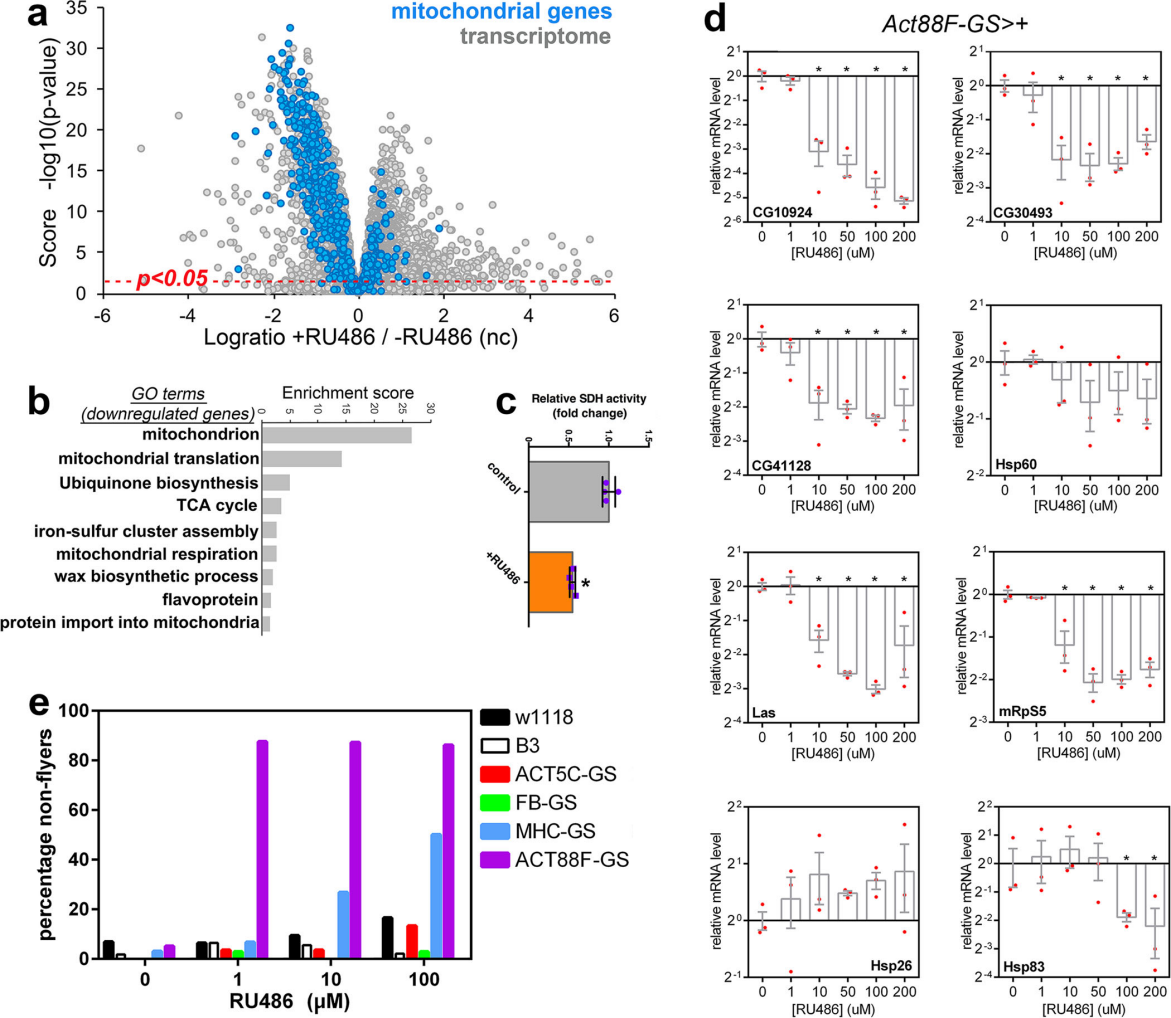
Transcriptional and functional defects induced by RU486 in skeletal muscle. **a** RNA-seq of thoracic skeletal muscle from *Drosophila Act88F-GS* flies treated with 100 µM RU486 for 10 days and untreated controls (–RU). RU486 represses the expression of nuclear-encoded mitochondrial genes, which is the first category of genes downregulated by RU486 (**b**). The transcriptome and the totality of mitochondrial genes are shown in gray and blue, respectively. The *x* axis reports the log ratio of gene expression changes of RU486-treated flies versus untreated controls, whereas the *y* axis reports the significance score, which is the –log10(*P*-value). Hence, values above the red line (*P* < 0.05) correspond to significantly regulated genes. No GO categories are identified in genes with RU486-upregulated expression. **c** RU486 treatment decreases SDH activity in the muscle of *Act88F-GS-Gal4* flies (*n* = 4, SD, *P < 0.05). **d** qRT-PCR analysis of skeletal muscle from *Act88-GS-Gal4* flies treated with RU486. Consistent with RNA-seq data, qPCR analysis confirms that the expression of mitochondrial genes (*CG10924*, *CG30493*, *CG41128*, *Hsp60*, *Las*, and *mRpS5*) declines in response to RU486 treatment in a dose-dependent manner. Conversely, RU486 induces the expression of the cytoplasmic chaperones *Hsp26* and *Hsp83* (*n* = 3, SD, *P < 0.05). **e** In flies with muscle expression of GS-Gal4 (*Mhc-GS-Gal4* and *Act88F-Gs-Gal4*), treatment for 1 week with 1, 10 or 100 µM RU486 leads to high percentages of flightless flies, compared with untreated controls. However, RU486 exerts minimal influence on the flight capacity of WT strains (*w*^*1118*^ and *B3*) and non-muscle GS-Gal4 strains with little or no GS-Gal4 expression in skeletal muscle (*Act5c-GS-Gal4*; *FB-GS-Gal4*, a combination of *S32-GS-Gal4* *+* *S106-GS-Gal4*). Supplementary Table 1 reports statistical analyses

However, in the course of these experiments, we observed serious unreported phenotypes that were induced by RU486. Specifically, by analyzing the gene expression changes induced by RU486 in the thoracic skeletal muscle of *Act88-GS-Gal4* flies (Supplementary Data 1), we found that RU486 represses the expression of nuclear-encoded mitochondrial genes (Fig. 1a). Consistent with this, several GO categories related to mitochondria were the most downregulated by RU486 (Fig. 1b). Moreover, the activity of succinate dehydrogenase (SDH), which is key for muscle function,^8^ was also decreased in thoracic skeletal muscle (Fig. 1c), suggesting that the RU486-induced decrease in mitochondrial gene expression also affects mitochondrial metabolism. Importantly, for these experiments, flies were reared for 10 days on 100 µM RU486, a concentration that is within the standard of up to 200 µM used in most studies.^1,9^ Subsequent qRT-PCR analyses of muscle samples from flies treated with different concentrations of RU486 indicate that defects in mitochondrial gene expression occur at fairly low levels (10 µM) of RU486 (Fig. 1d). These findings are in agreement with the effects of RU486 that have been observed in some mammalian cultured cells. Specifically, RU486 can inhibit mitochondrial gene expression through its antagonistic action on glucocorticoid receptors.^10–12^

**Fig. 2:**
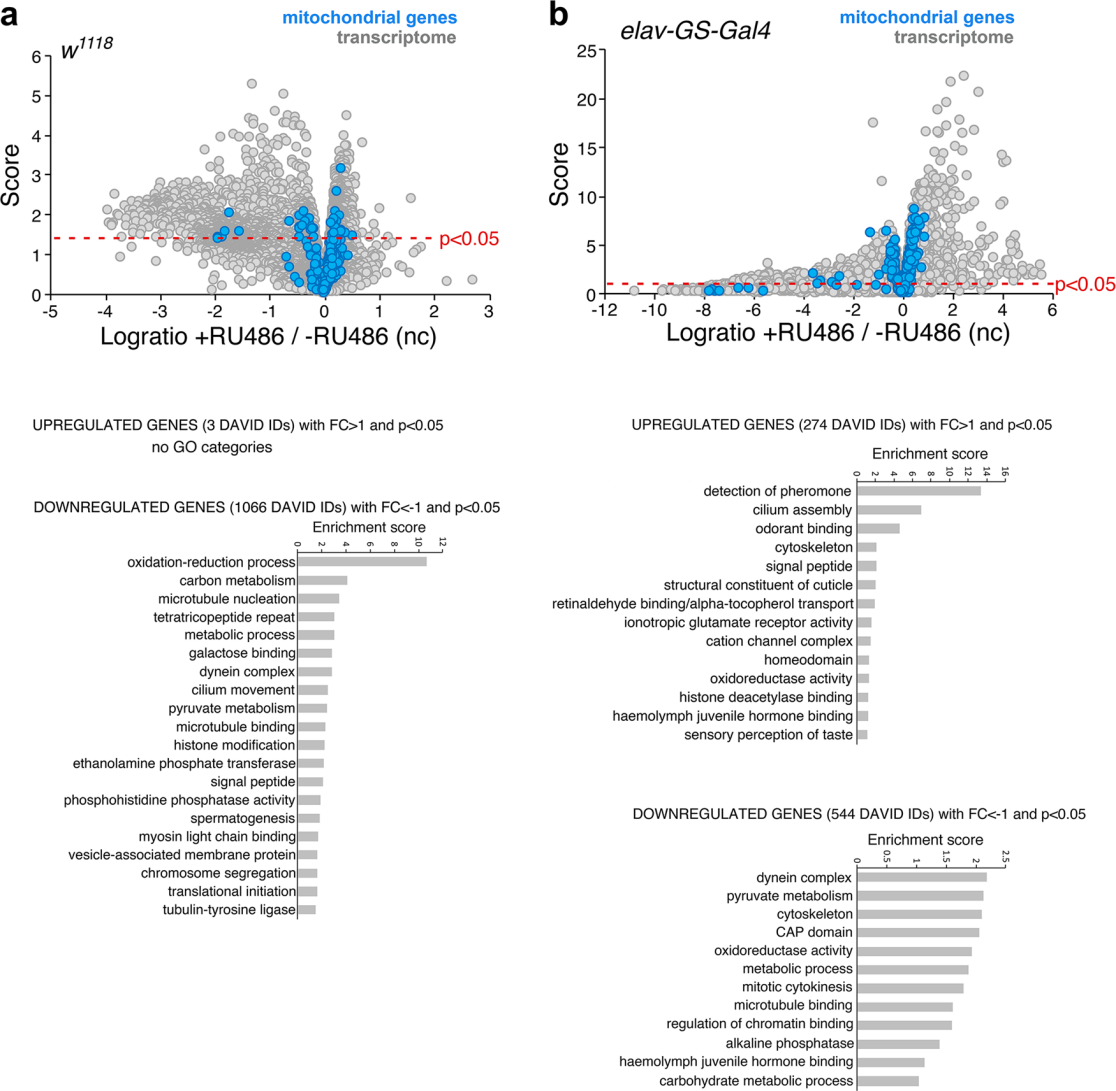
Tissue-specific changes induced by RU486 in a stock-dependent manner. **a** Treatment with 100 µM RU486 induces changes in gene expression in thoraces (consisting primarily of skeletal muscle) of *w*^*1118*^ flies. GO categories describing the 1066 genes that are downregulated with *p* < 0.05 and a Log2 ratio of <−1, compared with controls. Only three genes were upregulated with *p* < 0.05 and a Log2 ratio of >1. Only few mitochondrial genes (blue) are downregulated. **b** RU486-induced gene expression changes in head samples of *elav-GS-Gal4* flies. GO categories of genes transcriptionally modulated by treatment with 100 µM RU486. In total, 274 genes were upregulated with *p* < 0.05 and a Log2 ratio of >1, whereas 544 genes were downregulated with *p* < 0.05 and a Log2 ratio of <−1, compared with controls. Only few mitochondrial genes (blue) are downregulated

To further extend this analysis, we monitored the flight capacity of RU486-treated flies. We observed that a high percentage of flies expressing *Act88F-GS-Gal4* and *MHC-GS-Gal4* became flightless after RU486 treatment (Fig. 1e) at concentrations well below those used in most studies,^1,9^ presumably due to derangement of mitochondrial gene expression and function in flight muscles. However, only a minority of *w*^*1118*^ or *B3* wild-type flies and flies from non-muscle GS strains became flightless upon treatment with RU486 (Fig. 1e and Supplementary Table 1), suggesting that the interaction of RU486 with muscle-expressed GS-Gal4 affects flight ability. Interestingly, RU486 treatment of *w*^*1118*^ wild-type flies and of the neuronal-specific *elav-GS-Gal4* line did not affect the expression of mitochondrial genes in thoraces and heads, respectively, although the expression of many other gene categories was affected (Fig. 2 and Supplementary Data 1). These findings suggest that it is the interaction of RU486 with GS-Gal4 that deranges mitochondrial gene expression in *Drosophila* in a context-specific manner.

Altogether, these findings suggest a remarkable plasticity in the induction of gene expression changes by RU486, presumably in a manner that is dependent on the interaction of RU486 with the genetic background of a specific strain and characteristic features of a tissue, such as mitochondrial content.

## Discussion

In conclusion, the side effects described herein suggest that caution be taken when using RU486 and the GS system. We recommend that the side effects induced by RU486 should be evaluated in the context of the specific experimental settings. Side effects induced by RU486 vary in a stock- and tissue-dependent manner (Figs 1 and 2) and this should be taken into consideration when designing experiments and interpreting the observed phenotypes. For example, on the basis of these findings (Fig. 1), it seems inappropriate to use *Act88F-GS* as a tool to investigate pathways that regulate mitochondrial gene expression, as any effect due to pathway modulation may be masked by the effects that RU486 by itself has on mitochondrial gene expression.

Similarly, proper controls, consisting of ethanol-treated GS-Gal4 flies and RU486-induced expression of mock transgenes, should always be included to avoid erroneous conclusion about the effects of pathway modulation that may instead be explained by the action of RU486 by itself.

## Methods

### *Drosophila* husbandry and GeneSwitch-mediated expression

All experiments were performed with 10- to 14-day-old male flies kept (25–30 flies/tube) at 25 °C, 60% humidity, and a 12-h/12-h light–dark cycle. For experiments with the GS system, flies were raised for 4 days on normal food post eclosion and then kept for 10 days on food supplemented with the indicated concentration of RU486 (mifepristone; Calbiochem #475838) dissolved in ethanol, or with ethanol alone (control).

### Survival analysis

Lifespan experiments were done following standard procedures^13^ and by using 10 µM RU486 dissolved in ethanol to induce transgene expression. Ethanol alone was used as control treatment.

### Fly stocks

Fly stocks used in this study were *Act5c-GS-Gal4*, *WB-FB-GS-Gal4* (a combination of *S32-GS-Gal4* and *S106-GS-Gal4*), and *MHC-GS-Gal4*, which were described in a previous study.^6^ The *elav-GS-Gal4* (#43642) and *UAS-mCherry* (#35787) were obtained from the Bloomington *Drosophila* Stock Center. The wild-type strains used were *B3* and *w*^*1118*^. *UAS-foxo* fly stocks were also used.^7^

### Generation of *Act88F-GS-Gal4* fly stocks

*Act88F-GS-Gal4* was constructed by PCR-based amplification from genomic DNA of a 2-kb genomic region that includes the transcription start site of *Act88F*, using the same oligos used in a previous report:^14^ ACAATAGGCAAATTTAGTT and CATCTTGGCAGTTGTTTATC.

The PCR product was cut and cloned into the pCaSpeR5 vector with the EcoRI and KpnI enzymes. Subsequently, the open-reading frame that encodes GS-Gal4 was inserted downstream into KpnI/NotI sites. A SV40 poly(A) sequence was then inserted into the *Nhe*I site downstream of the GS-Gal4 sequence. The *pCaSpeR5-Act88F-GS-Gal4* plasmid was then injected into *yw* embryos, and fly stocks were established. A line with minimal leaky expression was then selected (Supplementary Figure 1).

### qRT-PCR

qRT-PCR was done as previously described.^13^ Specifically, total RNA was extracted with the TRIzol reagent (Life Technologies) from at least 20 thoraces from male flies per group, and reverse transcribed with the iScript cDNA synthesis kit (Bio-Rad). qRT-PCR was performed with the iQ SYBR Green Supermix and a CFX96 Real-Time PCR Detection System (Bio-Rad). *Alpha-Tubulin 84B* was used as a normalization reference. Relative quantitation of mRNA levels was done with the comparative C_T_ method. The following oligos were used:

*foxo*: 5′-CAGTGCCGGATGGAAGAACT-3′ and 5′-ATCCACCAGGATGACTTGCC-3′

*Hsp26*: 5′-ACGAGCTTGGACTGGGATTG-3′ and 5′-AAGGGCATCCGTTGATGGAA-3′

*Hsp83*: 5′-CGTTTGCCGGTTCGAGTCTT-3′ and 5′-TCTGCTTCTTCTGGCATCTTGT-3′

*Hsp60*: 5′-CCGTCGAAGAGGGAATCGTT-3′ and 5′-CACGCCGAGTTTCTGATCCT-3′

*mRpS5*: 5′-GCTTCTTTAACAAATTACCTGCTGA-3′ and 5′-TATGCGACCGCATTTTCCGA-3′

*Las*: 5′-TTAAGGCGCGCAACTCAAAC-3′ and 5′-TTGGCGATCGTCTTCACACA-3′

*CG30493*: 5′-GAGTTCCTCAAGAGCCGCAT-3′ and 5′-CGAGAATATTACGCGCCGTG-3′

*CG41128*: 5′-TTAAAGGATGTGGAGGCGTAA-3′ and 5′-CGTCCTATAAGCGATGCCCA-3′

*CG10924*: 5′-GGCCCAACAAAGCGCAATAA-3′ and 5′-TGGTGAGACCAATCCGCATC-3′

*AlphaTub84B*: 5′-GTTTGTCAAGCCTCATAGCCG-3′ and 5′-GGAAGTGTTTCACACGCGAC-3′

### SDH assays

For each sample, 15 thoraces were homogenized in a bullet blender (NextAdvance) with 100 µL of ice-cold SDH assay buffer, kept on ice for 10 min, and centrifuged at 10,000 × *g* for 5 min. Then, the supernatant was transferred to a fresh tube. In a 96-well plate, 25 µL of supernatant was plated per well, and the volume was adjusted to 50 µL with SDH assay buffer. Subsequently, 50 µL of a reaction mix (46 µL of SDH assay buffer, 2 µL of SDH assay mix, and 2 µL of SDH probe) was added to each well. The absorbance was immediately read at 600 nm in the kinetic mode for 25 min at 25 °C by using a Tecan Infinite M200 Pro microplate reader. The activity of SDH was calculated as per the manufacturer's instructions (Succinate Dehydrogenase kit, BioVision), as previously done.^15^

### RNA sequencing

After tissue homogenization with a bullet blender, RNA was extracted by using the TRIzol reagent (Life Technologies), and purified by using the RNeasy minikit (Qiagen) cleanup. Subsequently, samples were prepared for RNA-seq by using the TruSeq stranded mRNA library preparation kit (Illumina) and sequenced on the Illumina HiSeq 2000 platform. FastQ sequences were mapped to the *Drosophila* genome BDGP5.75 by StrongARM.^16^ Mapped reads were counted by using HTSeq,^17^ and gene-level fragments per kilobase per million mapped fragments (FPKM) values were computed. All sample data were collated into a matrix by using R (v3.0.1) and log start-transformed [log2(FPKM + 1] in STATA/MP 11.2. Genes were statistically tested by class (*t* test, unequal variance *t* test per gene). Bonferroni correction and false discovery rates (FDRs) were determined to allow both strict and modest multiple-comparison filtering (Partek Genomics suite 6.6). Significantly regulated genes were analyzed for enriched gene sets by using DAVID. Genes corresponding to *Drosophila* mitochondrial proteins annotated in the MitoMax database^18^ are highlighted in blue in Figs 1 and 2.

### Flight assays

Flight tests were done in a “Sparrow chamber”, an apparatus consisting of a plexiglass box that is 40-cm high and 20-cm wide, with a small opening to introduce the flies and having a light source at the top.^19^ Flight tests were performed by releasing each fly to the center of the box and then scoring its ability to fly. Flies were defined as non-flyers if they fall at the bottom at the chamber.

### Statistical analyses

All experiments were performed with biological triplicates unless otherwise indicated. The unpaired two-tailed Student's *t* test was used to compare the means of two independent groups to each other. ANOVA with Tukey's post hoc test was used for multiple comparisons of more than two groups of normally distributed data. Flight assays were analyzed with the chi-square analysis of contingency tables and Fisher exact tests. The *n* for each experiment can be found in the figure or figure legends and represents individual flies, batches of flies, and of batches of fly tissues for all in vivo experiments, as indicated in the figure legends and Methods. Bar graphs represent the mean ± SD. A result was defined as significant if *P < 0.05. Statistical analyses were done with Excel and GraphPad Prism. Statistical analysis of lifespan data was done using OASIS^20^ with Fisher exact test at 50% survival.

## Supplementary information

Supplementary Information

## Data Availability

The RNA-sequencing data sets that support the findings of this study are provided in Supplementary Data 1 and are available at the Gene Expression Omnibus (GSE129815). Flight assay results are provided in Supplementary Table 1. Other data sets are available from the corresponding author upon request.

## Supplementary information

**Supplementary Information** accompanies the paper on the *npj Aging and Mechanisms of Disease* website (10.1038/s41514-019-0036-8).
