# Supplementary material for: Tissue-specific alteration of gene expression and function by RU486 and the GeneSwitch system
Source: NPJ Aging Mech Dis. 2019 May 21;5:6. doi: 10.1038/s41514-019-0036-8 (PMC6529481; doi:10.1038/s41514-019-0036-8)
Supplement: Supplementary file 1 — Supplementary Information [file 41514_2019_36_MOESM1_ESM.pdf]

SUPPLEMENTARY INFORMATION (Robles-Murguia et al.)

Tissue-specific alteration of gene expression and function by RU486 and the GeneSwitch system

SUPPLEMENTARY FIGURES

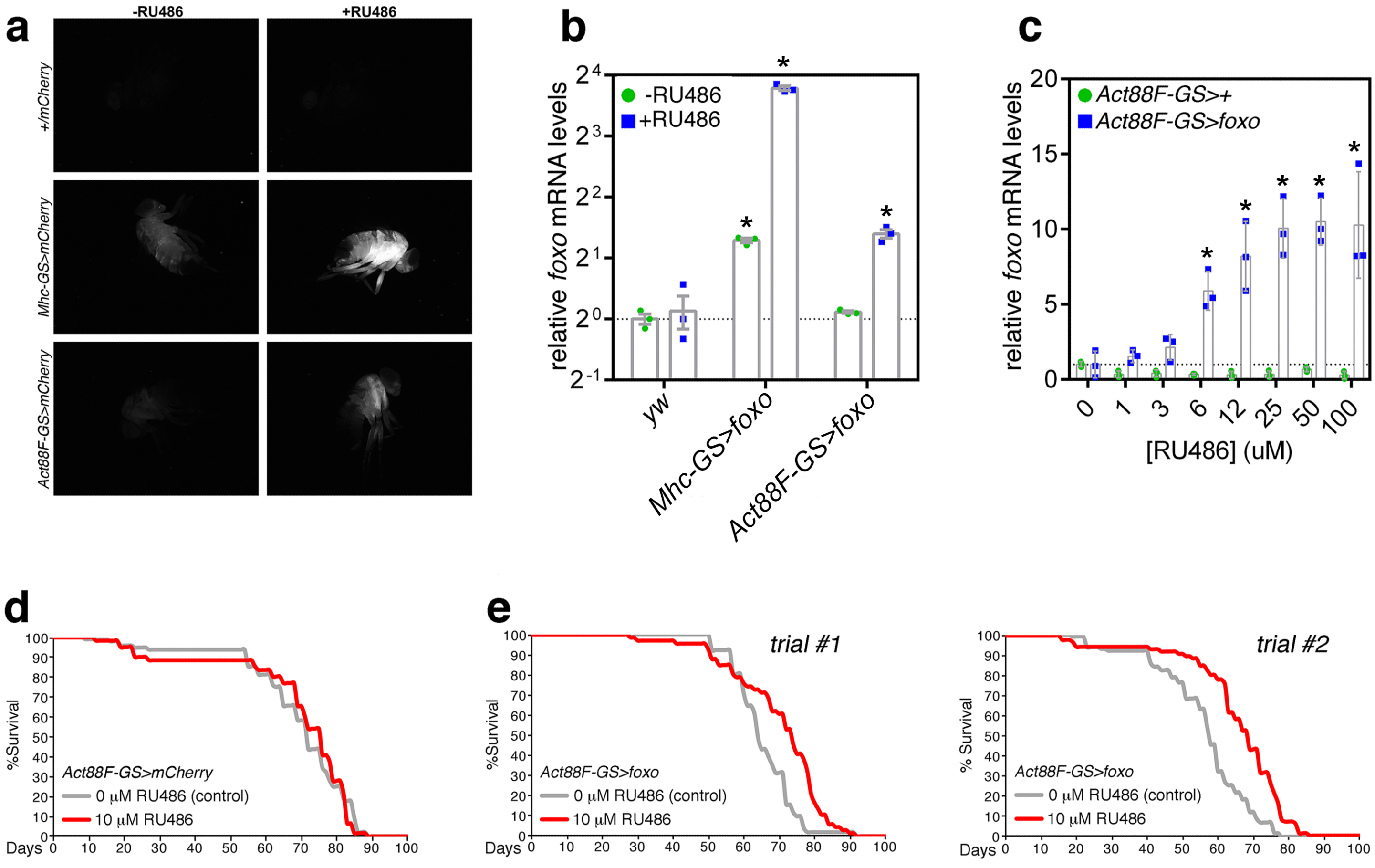

Supplementary Figure 1. Characterization of the *Act88F-GS-Gal4* driver for drug-induced transgenic expression in *Drosophila*. **a** Compared to the *Mhc-GS-Gal4*, which is leaky[1], *Act88F-GS-Gal4* drives expression of *mCherry* transgenes only in presence of RU486 but not in its absence. As inferred from the fluorescence of *mCherry* transgenes, *Act88F-GS-Gal4* drives transgenic expression specifically in thoracic flight muscles and to a lesser extent in leg muscles. No expression is detected in non-muscle tissues. **b** Consistently, *foxo* expression is induced by *Act88F-GS-Gal4* only in presence of RU486 but not in its absence, whereas leaky *foxo* expression occurs with *Mhc-GS-Gal4* even in the absence of RU486. **c** Transgenic *foxo* expression with *Act88F-GS-Gal4* is dose-dependent. In **b-c**, n=3 and the error bars represent the SD.

d *mCherry* expression induced with 10 μM RU486 and *Act88F-GS-Gal4* does not extend lifespan (P=0.25; n[control]=72 and n[RU486]=76). **e** RU486-induced overexpression of wild-type *foxo* with *Act88F-GS-Gal4* significantly extends lifespan (trial #1: P=0.0014; n[control]=64 and n[RU486]=74); trial #2: P<0.0001; n[control]=58 and n[RU486]=69), consistent with previous results[2], obtained with a constitutive *Mhc-Gal4* driver.

SUPPLEMENTARY REFERENCES

1 Poirier, L., Shane, A., Zheng, J. & Seroude, L. Characterization of the Drosophila gene-switch system in aging studies: a cautionary tale. *Aging Cell* **7**, 758-770, doi:10.1111/j.1474-9726.2008.00421.x (2008).

2 Demontis, F. & Perrimon, N. FOXO/4E-BP signaling in Drosophila muscles regulates organism-wide proteostasis during aging. *Cell* **143**, 813-825, doi:10.1016/j.cell.2010.10.007 (2010).

SUPPLEMENTARY TABLES

	Control		
WT strains	Non-flyers	Flyers	Percentage
w1118	8	109	6.84
B3	2	112	1.75
GeneSwitch strains			
ACT5C	0	66	0.00
WB-FB	0	85	0.00
MHC	3	59	4.84
ACT88F	5	79	5.95

	1uM RU486				
WT strains	Non-flyers	Flyers	Percentage	Fisher P=	Chi+Yates P=
w1118	8	117	6.40	1.0000	0.8911
B3	9	130	6.47	0.1179	0.1280
GeneSwitch strains					
ACT5C	4	60	6.25	0.0559	0.1199
WB-FB	4	92	4.17	0.1236	0.1626
MHC	2	48	4.00	1.0000	0.8308
ACT88F	61	8	88.41	0.0001	0.0001

	10uM RU486				
WT strains	Non-flyers	Flyers	Percentage	Fisher P=	Chi+Yates P=
w1118	14	135	9.40	0.5076	0.5977
B3	6	103	5.50	0.1636	0.2522
GeneSwitch strains					
ACT5C	6	56	9.68	0.0113	0.0300
WB-FB	1	87	1.14	1.0000	0.3243
MHC	11	58	15.94	0.0492	0.0766
ACT88F	73	5	93.59	0.0001	0.0001

	100uM RU486				
WT strains	Non-flyers	Flyers	Percentage	Fisher P=	Chi+Yates P=
w1118	23	116	16.55	0.0207	0.0293
B3	3	138	2.13	1.0000	0.8307
GeneSwitch strains					
ACT5C	4	61	6.15	0.0578	0.1238
WB-FB	2	89	2.20	0.4977	0.5073
MHC	33	42	44.00	0.0001	0.0001
ACT88F	76	5	93.83	0.0001	0.0001

Supplementary Table 1. Statistical analysis of flight assays.
